# Immune response profiling in early rheumatoid arthritis: discovery of a novel interaction of treatment response with viral immunity

**DOI:** 10.1186/ar4389

**Published:** 2013-11-25

**Authors:** John M Davis, Keith L Knutson, Michael A Strausbauch, Abigail B Green, Cynthia S Crowson, Terry M Therneau, Eric L Matteson, Sherine E Gabriel

**Affiliations:** 1 Division of Rheumatology, Department of Medicine, College of Medicine, Mayo Clinic, 200 First Street SW, Rochester, MN 55905, USA; 2 Cancer Vaccine and Immune Therapies Program, Vaccine & Gene Therapy Institute of Florida, 9801 S.W. Discovery Way, Port Saint Lucie, FL 34987, USA; 3 Department of Surgical Research, College of Medicine, Mayo Clinic, 200 First Street SW, Rochester, MN 55905, USA; 4 Division of Biomedical Statistics and Informatics, Department of Health Sciences Research, College of Medicine, Mayo Clinic, 200 First Street SW, Rochester, MN 55905, USA; 5 Division of Epidemiology, Department of Health Sciences Research, College of Medicine, Mayo Clinic, 200 First Street SW, Rochester, MN 55905, USA

## Abstract

**Introduction:**

It remains challenging to predict the outcomes of therapy in patients with rheumatoid arthritis (RA). The objective of this study was to identify immune response signatures that correlate with clinical treatment outcomes in patients with RA.

**Methods:**

A cohort of 71 consecutive patients with early RA starting treatment with disease-modifying antirheumatic drugs (DMARDs) was recruited. Disease activity at baseline and after 21 to 24 weeks of follow-up was measured using the Disease Activity Score in 28 joints (DAS28). Immune response profiling was performed by analyzing multi-cytokine production from peripheral blood cells following incubation with a panel of stimuli, including a mixture of human cytomegalovirus (CMV) and Epstein-Barr virus (EBV) lysates. Profiles identified via principal components analysis (PCA) for each stimulus were then correlated with the ΔDAS28 from baseline to follow-up. A clinically meaningful improvement in the DAS28 was defined as a decrease of ≥1.2.

**Results:**

A profile of T-cell cytokines (IL-13, IL-4, IL-5, IL-2, IL-12, and IFN-γ) produced in response to CMV/EBV was found to correlate with the ΔDAS28 from baseline to follow-up. At baseline, a higher magnitude of the CMV/EBV immune response profile predicted inadequate DAS28 improvement (mean PCA-1 scores: 65.6 versus 50.2; *P* = 0.029). The baseline CMV/EBV response was particularly driven by IFN-γ (*P* = 0.039) and IL-4 (*P* = 0.027). Among patients who attained clinically meaningful DAS28 improvement, the CMV/EBV PCA-1 score increased from baseline to follow-up (mean +11.6, SD 25.5), whereas among patients who responded inadequately to DMARD therapy, the CMV/EBV PCA-1 score decreased (mean -12.8, SD 25.4; *P* = 0.002). Irrespective of the ΔDAS28, methotrexate use was associated with up-regulation of the CMV/EBV response. The CMV/EBV profile was associated with positive CMV IgG (*P* <0.001), but not EBV IgG (*P* = 0.32), suggesting this response was related to CMV exposure.

**Conclusions:**

A profile of T-cell immunity associated with CMV exposure influences the clinical response to DMARD therapy in patients with early RA. Because CMV latency is associated with greater joint destruction, our findings suggest that changes in T-cell immunity mediated by viral persistence may affect treatment response and possibly long-term outcomes of RA.

## Introduction

The ideal approach for predicting and monitoring the outcomes of treatment in rheumatoid arthritis (RA) remains elusive. The advent of intensive, goal-directed treatment strategies, employing combinations of synthetic as well as biologic disease-modifying antirheumatic drugs (DMARDs), has substantially improved the prognosis of this disease [1]. However, many patients still fail to achieve low disease activity or remission [2-4]. Failure to completely abrogate inflammation puts patients at risk for disease progression, including joint destruction, disability, impaired quality of life, cardiovascular disease, and premature death [5,6].

The complexity of this issue emerges with the realization that, at times, discriminating clinical signs and symptoms of active joint inflammation from non-inflammatory joint disease or chronic pain syndromes is challenging [7-9]. With many synthetic and biologic DMARDs now available, our limited ability to predict and to efficiently judge the likely outcomes of DMARD therapy represents a critical barrier to the development of more effective treatment strategies using these agents.

Recently, we have established an approach of immune-response profiling for the discovery of complex biomarkers predictive of treatment response [10]. Although the levels of serum cytokines have proven to be disappointing for use as disease biomarkers [10-14], our work has suggested that immune response signatures may provide greater biologically relevant information. The results of our published studies demonstrate proof of principle that our approach, based on multiplexed analysis of *ex vivo* cytokine production in response to broad stimulation, can identify profiles of immune function associated with radiographic joint damage as well as myocardial disease in patients with RA [10,15]. Further, the discovery of a correlation between a profile of immune response to Epstein-Barr virus (EBV) and human cytomegalovirus (CMV) and the severity of radiographic joint destruction in RA demonstrates the relevance of the data generated to the investigation of pathogenesis [16]. The purpose of this study was to identify immune response signatures that are associated with treatment outcomes in patients with early RA.

## Methods

### Study design and participants

A 24-week prospective observational cohort study of patients with newly diagnosed RA was performed at our institution. Consecutive patients with a new diagnosis of inflammatory arthritis during the enrollment period of July 2008 to December 2010 were referred for screening by rheumatologists in our division. At conception of this study, the available classification criteria for RA were the 1987 criteria, which were too insensitive in early disease for use in this study (which took place before the 2010 revised classification criteria were published) [17]. Therefore, the Leiden early RA prediction rule was used [18,19]. Eligible patients were required to have a score ≥8 on the early RA prediction rule and to be starting their initial treatment with conventional DMARDs within 3 weeks of diagnosis. Patients who were prescribed biologic agents were ineligible. All patients (n = 71) participated in research study visits at baseline and after 21 to 24 weeks of follow up. Patients were required to provide written informed consent prior to study participation. The institutional review board of the Mayo Foundation approved this study.

### Data collection

During each research study visit, one consultant rheumatologist (JMD) performed a standardized clinical evaluation of the patient, consisting of the 68-tender joint count, the 66-swollen joint count and the physician global assessment (0 to 100 mm). All patients completed the study questionnaire, which included visual analog scales (0 to 100 mm) for the levels of pain, fatigue and the patient global assessment of disease activity, and the Health Assessment Questionnaire (HAQ) disability index [20,21]. The dates of initiation/change, dosages, and route (that is, oral or intra-articular) of DMARDs or corticosteroids were obtained from the patients and confirmed in the most recent outpatient medication list. Medical records were reviewed to collect relevant demographic information, symptom duration at baseline, body mass index (kg/m^2^), smoking status (current, former, or never), and test results for rheumatoid factor (RF) and anti-citrullinated protein antibodies (ACPA). C-reactive protein (CRP) was measured by turbidometric assay (Roche Diagnostics, Indianapolis, Indiana, USA). Enzyme-linked immunosorbent assays were done to assess past CMV infection or exposure using the VIDAS® CMV IgG (bioMerieux, Inc., Marcy l'Etoile, France), and multiplexed immunoassays were done to assess past EBV infection using the BioPlex™ 2200 System assessing EBV IgG and IgM (Bio-Rad Laboratories, Hercules, California, USA).

### Definition of outcome measures

Clinical disease activity was defined by the Disease Activity Score in 28 joints (DAS28), based on the four-variable version using CRP [22,23]. Physical disability was defined by the HAQ disability index. Treatment response was defined as the difference (Δ) between the baseline and 21- to 24-week values for the DAS28 and HAQ disability index. A clinically meaningful improvement in the DAS28 was defined as a decrease ≥1.2.

### Immune-response profiling

Immune-response profiling was performed on samples obtained from all 71 patients at the baseline visit and from a subset of 43 patients at the 21- to 24-week visit. Detailed methods of our approach to profiling the systemic immune response were previously described [10]. Briefly, peripheral blood mononuclear cells (PBMC) from patients were cultured in the presence of a panel of six stimuli, or in media alone, for 48 hours. Stimuli used were immobilized anti-CD3/anti-CD28 monoclonal antibodies (anti-CD3/anti-CD28), combined lysates of purified CMV and EBV, containing both viral peptides and DNA, phytohemagglutinin (PHA), phorbol myristate acetate with ionomycin (PMA/ionomycin), a mixture of staphylococcal enterotoxins A and B (SEA/SEB), and CpG oligonucleotides. At 48 hours of culture, cell-free supernatants were removed and frozen for subsequent cytokine analysis.

The production of cytokines in the supernatants was analyzed using the MSD® 96-well MULTI-SPOT® Human Cytokine Assays tissue culture kit (Meso Scale Discovery (MSD), Rockville, Maryland, USA). The cytokine panel included IL-1β, IL-2, IL-4, IL-5, IL-6, IL-7, IL-8 (CXCL8), IL-10, IL-12, IL-13, IL-17A, IFN-γ, TNF-α, monocyte chemoattractant protein (MCP)-1 (CCL2), monocyte inflammatory protein (MIP)-1β (CCL4), granulocyte colony stimulating factor (G-CSF), and granulocyte monocyte colony stimulating factor (GM-CSF).

### Statistical analysis

The baseline characteristics were analyzed using descriptive statistics, including mean (SD) or median (range) as appropriate. All statistical tests were two-sided; the significance level was set at 0.05 for all analyses. Paired *t*-tests were used to assess changes in characteristics between baseline and follow-up visits. Mixed models were used to normalize the cytokine data and adjust for age and sex as previously described [10]. Principal components analysis (PCA) was used to derive immune-response profiles based on the first and second principal components of *ex vivo* cytokine production for each stimulus [16]. This analytic technique provided a relative weighting of cytokine importance in each profile and quantitatively summarized the information in immune-response profiles as PCA scores (rescaled 0 to 100 for interpretation) for subsequent screening.

Spearman methods and Wilcoxon rank sum tests were used to test for associations of both the baseline immune response PCA scores and the changes in these scores from baseline to 21 to 24 weeks with the changes in the clinical disease activity measures from baseline to 21 to 24 weeks. For immune profiles significantly associated with treatment response, partial Spearman correlation was used to adjust for potential confounding factors, including age, sex, body mass index, smoking status, RF status, ACPA status, CMV immunoglobulin (Ig)G, EBV IgG, methotrexate use, and prednisone use.

## Results

### Baseline patient characteristics

A total of 71 patients with early RA according to the Leiden early RA prediction rule were recruited. Of these, 39 (55%) also fulfilled the 1987 American College of Rheumatology (ACR) classification criteria. The mean (SD) age of the cohort was 56 (13) years, and 47 (66%) were female (Table 1). The median duration of symptoms was 5.5 months (range 0.5 to 45.5). About 90% were ACPA positive. At baseline, the mean (SD) DAS28 was 4.9 (1.0), consistent with moderately high disease-activity, and the mean (SD) HAQ disability index was 0.9 (0.5), corresponding to mild-to-moderate disability. At the baseline visit, 27 (38%) patients had already taken their first dose of oral DMARDs, and 16 (23%) were on prednisone (Table 2). The remainder of patients began DMARDs and/or prednisone after the initial study blood draw.

**Table 1:** Characteristics at baseline and follow up of the 71 patients with early RA

**Variable**	**Baseline**	**Follow up**	***P***
Age	56.4 (12.7)	-	-
Female	47 (66%)	-	-
Duration of symptoms, months	10.3 (11.6)	-	-
Smoking status (current)	15 (21%)	-	-
Body mass index (kg/m^2^)	30.6 (7.2)	-	-
Rheumatoid factor-positive	55 (79%)	-	-
ACPA-positive	63 (89%)	-	-
CMV-IgG-positive	30 (42%)	-	-
EBV-IgG-positive	64 (90%)	-	-
DAS28-CRP (four-variable)	4.9 (1.0)	3.6 (1.3)	<0.001
HAQ disability index	0.9 (0.5)	0.5 (0.6)	<0.001
C-reactive protein (mg/L)	12.6 (18.0)	6.3 (7.9)	0.003

Data are mean (SD) or number (%) as appropriate. RA, rheumatoid arthritis; ACPA, anti-citrullinated protein antibodies; DAS28, Disease Activity Score in 28 joints; HAQ, Health Assessment Questionnaire; CMV, cytomegalovirus; EBV, Epstein-Barr virus; DMARD, disease-modifying antirheumatic drug.

**Table 2:** Characteristics of exposure to DMARDs during follow up among 71 patients with early RA

**Medication**	**Baseline**		**Follow up**	
	**Prior to baseline**	**Prescribed at baseline visit**	**Cumulative exposure**	**Taking at follow-up visit**
Methotrexate	14 (20%)	49 (69%)	51 (72%)	50 (70%)
Hydroxychloroquine	8 (11%)	24 (34%)	44 (39%)	28 (39%)
Combination DMARD	5 (7%)	10 (14%)	17 (24%)	16 (23%)
Prednisone	16 (23%)	46 (65%)	49 (69%)	29 (41%)
Dose, mean ± SD		17.4 ± 14.2		6.7 ± 5.6
Median (minimum, maximum)		10.0 (5, 75)		5 (1, 25)

Medications prescribed at the baseline visit include initiation within 2 weeks after baseline. DMARD, disease-modifying antirheumatic drug.

Initial DMARD therapy was prescribed by each patient’s primary rheumatologist: 49 patients (69%) received single-drug therapy with methotrexate, 24 (34%) received hydroxychloroquine, 10 (14%) received double- or triple-DMARD combination therapy, and 4 (6%) received other DMARDs (Table 2). Forty-six patients (65%) were also treated initially with prednisone. Treatment with DMARDs and prednisone during the study is shown in Table 2.

By follow up at 21 to 24 weeks, significant clinical improvements were noted (Table 1). The median ΔDAS28 was -1.1 (range -4.4, 0.7), and the median ΔHAQ disability index was -0.5 (range -2.1, 1.0). Significant predictors of the ΔDAS28 from baseline to follow up were age, baseline DAS28 and smoking status (data not shown).

### Discovery of immune response profiles

Profiles of cytokine release from PBMC into culture supernatants in response to the various stimuli were identified using PCA analysis (Table 3). For CMV/EBV stimulation, PCA of the first principal component (PCA-1) revealed an immune response profile consisting of type 1 and type 2 T-cell cytokines. Selecting cytokines with PCA weightings >0.5, the profile (ordered from high to low weightings) included IL-13, IL-4, IL-5, IL-2, IL-12, and IFN-γ. The immune response profile for PHA similarly reflected a T-cell response, comprising IL-4, IL-5, IL-10, IL-13, IFN-γ, IL-12, MIP-1β, TNF-α, and IL-2. Using the same selection criteria, the profile of basal cytokine production in media alone for the second principal component (PCA-2) included IL-1β, IFN-γ, G-CSF, TNF-α, and IL-6.

**Table 3:** **Principal components analysis identifies immune response profiles defined by multiplexed detection of *ex vivo *** **cytokine production in 71 patients with early RA**

**Cytokine**	**CD3/CD28**		**CMV/EBV**		**CpG**		**PHA**		**PMA**		**SEA/SEB**		**Media**	
G-CSF	-0.158	0.112	-0.224	0.540	0.736	-0.095	-	0.933	0.802	-0.131	0.883	0.085	0.334	0.564
GM-CSF	0.202	0.795	0.186	0.828	0.438	-0.005	0.300	0.225	0.700	0.343	0.706	0.388	0.829	0.111
IFN-γ	0.709	0.101	0.690	0.176	0.495	0.013	0.708	0.523	0.144	0.283	0.061	0.613	0.196	0.623
IL-1β	0.178	0.202	0.086	0.306	0.722	-0.116	0.224	0.722	0.568	0.093	0.609	0.561	0.196	0.685
IL-2	0.693	0.298	0.798	0.211	-0.194	0.291	0.586	0.089	0.136	0.418	0.243	0.422	0.167	-0.055
IL-4	0.783	0.103	0.898	0.152	-0.055	0.932	0.892	0.165	-0.047	0.647	0.020	-0.040	-0.069	-0.131
IL-5	0.189	0.184	0.858	0.104	-0.083	0.739	0.825	0.260	-0.005	0.207	0.095	0.217	-0.138	-0.029
IL-6	0.101	0.716	-0.320	0.495	0.632	-0.107	0.298	0.820	0.772	0.012	0.827	0.319	0.642	0.532
IL-7	0.154	0.181	0.013	-0.102	-0.018	0.051	0.141	0.343	0.349	0.076	0.030	0.055	0.133	0.030
IL-8	0.177	-0.502	-0.117	-0.386	-0.727	0.146	-0.233	-0.748	-0.925	0.088	-0.480	-0.656	-0.151	-0.394
IL-10	0.830	0.152	0.328	-0.142	0.085	0.075	0.790	0.320	-0.006	0.469	0.145	0.060	0.121	0.130
IL-12	0.755	-0.174	0.787	0.110	-0.216	0.880	0.686	0.009	-0.058	0.800	-0.051	-0.004	-0.098	-0.128
IL-13	0.377	0.254	0.908	0.159	0.104	0.413	0.777	0.375	0.316	0.209	0.170	0.038	0.036	0.025
IL-17	0.157	0.786	0.369	0.787	0.024	0.073	0.247	0.054	0.283	0.207	0.841	0.175	0.612	0.054
MCP-1	-0.703	0.311	0.305	0.759	0.510	-0.109	0.252	0.650	0.682	-0.067	0.798	0.047	0.775	0.253
MIP-1β	0.484	0.247	0.100	0.444	0.543	-0.010	0.681	0.519	0.147	0.660	0.362	0.376	0.591	0.357
TNF-α	0.765	0.424	0.268	0.159	0.805	0.018	0.661	0.584	0.768	0.448	0.228	0.750	0.600	0.539

Shown are the loadings for each cytokine in the first (left column for each stimulus) and the second (right column for each stimulus) principal components. CMV, cytomegalovirus; EBV, Epstein-Barr virus; CpG, CpG oligonucleotides; PHA, phytohemagglutinin; PMA, phorbol myristate acetate with ionomycin; SEA/SEB, staphylococcal enterotoxins A and B; G-CSF, granulocyte colony stimulating factor; GM-CSF, granulocyte monocyte colony stimulating factor; IFN, interferon; IL, interleukin; MCP-1, monocyte chemoattractant protein 1; MIP-1β, monocyte inflammatory protein 1-beta; TNF-α, tumor necrosis factor-α.

### Correlation of the baseline immune response profiles with treatment outcomes

The next step was to screen the immune response profiles identified at baseline for correlation with the clinical outcomes at follow up (Table 4). The baseline CMV/EBV PCA-1 score (*r* = 0.38, *P* = 0.007) and the baseline media-alone PCA-2 score (*r* = 0.31, *P* = 0.034) were found to correlate significantly with the ΔDAS28, after adjusting for clinical covariates, including age, sex, body mass index, smoking status, RF status, ACPA status, CMV IgG status, methotrexate use, and prednisone use. However, correlations of both CMV/EBV PCA-1 and media-alone PCA-2 with ΔHAQ did not reach statistical significance.

**Table 4:** Association of the baseline immune-response profiles with the clinical outcomes of initial therapy at 21 to 24 weeks of follow up in 71 patients with RA

		**ΔDAS28**		**ΔHAQ-DI**	
**Profile**	**Model**	**r**	***P***	**r**	***P***
CMV/EBV-1	Unadjusted	0.28^b^	0.03^b^	-0.05	0.65
	Adjusted^a^	0.38^b^	0.007^b^	0.08	0.59
CMV/EBV-2	Unadjusted	-0.01	0.94	-0.18	0.13
	Adjusted^a^	0.04	0.79	-0.13	0.36
PHA-1	Unadjusted	0.03	0.81	0.03	0.80
	Adjusted^a^	0.02	0.91	-0.01	0.93
PHA-2	Unadjusted	0.16	0.23	0.10	0.42
	Adjusted^a^	0.13	0.40	0.09	0.56
Media-1	Unadjusted	0.22	0.09	0.10	0.41
	Adjusted^a^	0.27	0.064	0.19	0.20
Media-2	Unadjusted	0.26^b^	0.04^b^	0.12	0.33
	Adjusted^a^	0.31^b^	0.034^b^	0.19	0.20

Shown are the Spearman correlations between the PCA stimulus-cytokine scores for the first and second principal components and both the change in the DAS28 and the change in the HAQ disability index from baseline to 21 to 24 weeks. ^a^Adjusted for age, sex, body mass index, smoking status, RF status, ACPA status, CMV IgG status, methotrexate use, and prednisone use; ^b^values are statistically significant. DAS28, Disease Activity Score in 28 joints; HAQ-DI, Health Assessment Questionnaire disability index; CMV/EBV, cytomegalovirus/Epstein-Barr virus; PHA, phytohemagglutinin; RF, rheumatoid factor; ACPA, anti-citrullinated protein antibodies.

**Figure 1:**
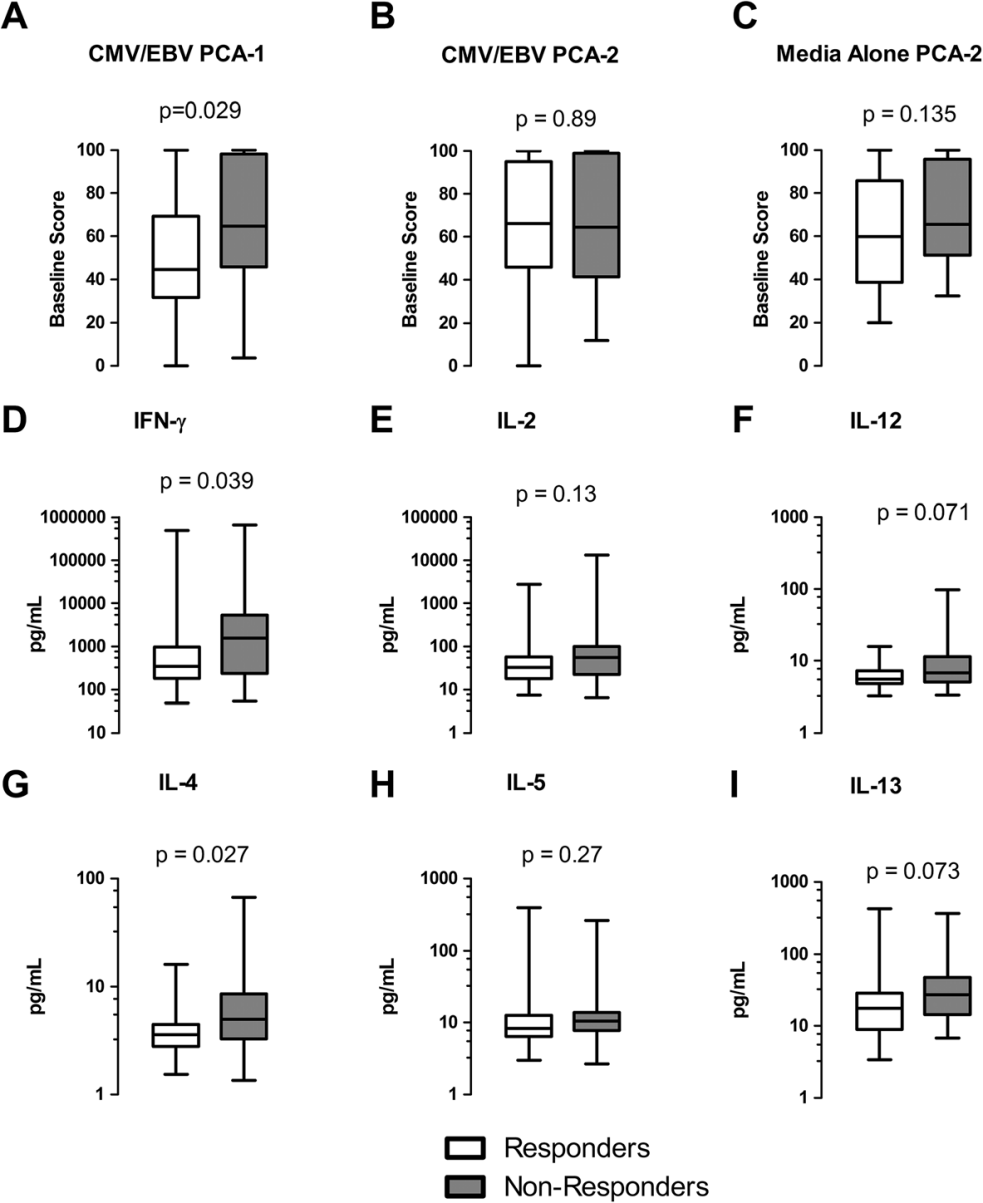
**Association between the baseline immune response signatures for cytomegalovirus/Epstein-Barr virus (CMV/EBV) and media alone and clinical improvement at 21 to 24 weeks among 71 patients with early rheumatoid arthritis. (A-C)** Shown are box plots for the distributions of the CMV/EBV PCA-1, CMV/EBV PCA-2, and media alone PCA-2 scores, respectively, comparing responders and non-responders to initial disease-modifying treatment. Values were scaled from 0 to 100. **(D-I)** Shown are box plots representing the distributions of individual cytokines in the CMV/EBV PCA-1 signature in pg/mL. Clinical response was defined by a decrease in the DAS28 ≥1.2 from baseline to follow up. PCA, principal components analysis; DAS28, disease activity score in 28 joints.

As shown in Figure 1A, patients who failed to achieve clinically meaningful DAS28 improvement had a significantly higher CMV/EBV PCA-1 score at baseline than patients who responded well to DMARD therapy (mean 65.6 versus 50.2, *P* = 0.029). Higher CMV/EBV PCA-1 scores in non-responders were driven mainly by increased CMV/EBV-induced IFN-γ (Figure 1D) and IL-4 (Figure 1G). The baseline scores for CMV/EBV PCA-2 and media alone PCA-2 were similar between responders and non-responders (Figure 1B-C).

Of note, significant correlation with treatment outcome was not found for the immune response profiles of CPG or PHA (Table 4), or any of the other stimuli (data not shown).

### Correlation of the changes in immune response profiles from baseline to follow up with changes in clinical disease status

**Figure 2:**
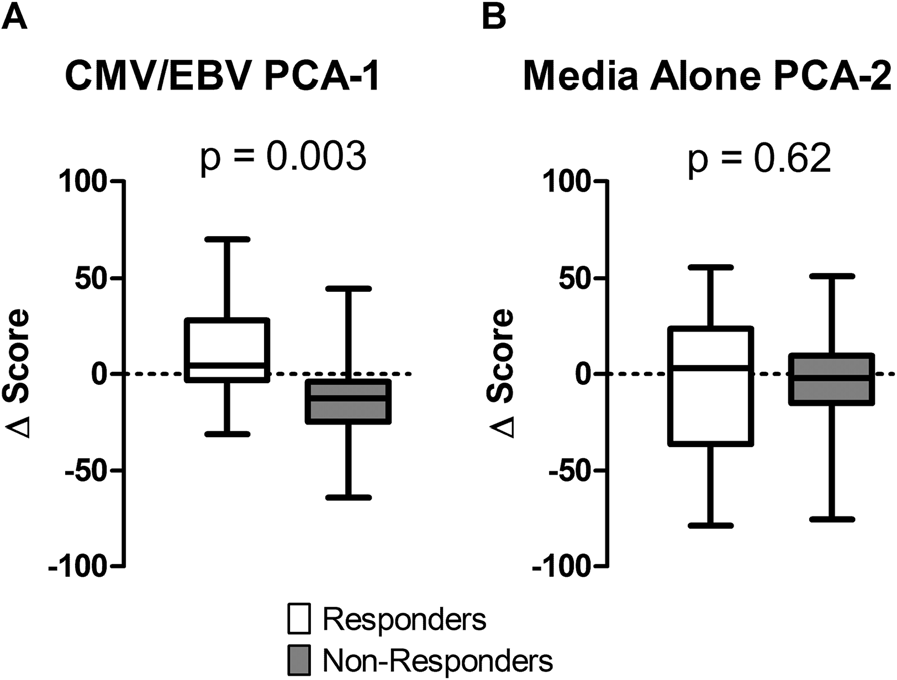
**Associations between the changes in the cytomegalovirus/Epstein-Barr virus (CMV/EBV) and media-alone immune response signatures from baseline to 21 to 24 weeks and clinical response to initial disease-modifying antirheumatic drug (DMARD) therapy among 43 patients with early rheumatoid arthritis.** Shown are box plots representing the distributions of the changes from baseline to 21 to 24 weeks in **(A)** the CMV/EBV PCA-1 scores and **(B)** the media-alone PCA-2 scores, comparing responders and non-responders. Clinical response was defined as a decrease in the DAS28 ≥1.2 from baseline to 21 to 24 weeks. The horizontal dotted lines represent the medians. PCA, principal components analysis; DAS28, Disease Activity Score using 28 joints.

The subsequent analysis tested correlation between the changes in the immune response profiles and the changes in clinical disease activity and disability from baseline to follow up in a subset of 43 patients with complete data at both visits (Table 5). The change in the CMV/EBV PCA-1 score was found to correlate significantly with the ΔDAS28 from baseline to follow-up (*r* = -0.39, *P* = 0.032), after adjusting for clinical covariates. As shown in Figure 2A, among patients who attained clinically meaningful DAS28 improvement, the mean CMV/EBV PCA-1 score increased from baseline to follow up (mean +11.6, SD 25.5), whereas among patients who responded inadequately to DMARD therapy, the CMV/EBV PCA-1 score decreased (mean -12.8, SD 25.4; *P* = 0.002). Among patients who experienced augmentation of their CMV/EBV immune response, this effect was consistent across all of the six T-cell cytokines (data not shown). Similar results were observed for the change in HAQ disability index, underscoring the robustness of these results.

**Table 5:** Association of the changes in the immune response profiles with the changes in clinical disease activity and disability from baseline to 21 to 24 weeks of follow up in 43 patients with RA

		**ΔDAS28**		**ΔHAQ-DI**	
**Profile**	**Model**	***r***	***P***	***r***	***P***
CMV/EBV-1	Unadjusted	-0.40^b^	0.01^b^	-0.36^b^	0.02^b^
	Adjusted^a^	-0.39^b^	0.032^b^	-0.39^b^	0.031^b^
CMV/EBV-2	Unadjusted	-0.03	0.87	-0.15	0.33
	Adjusted^a^	0.07	0.72	-0.10	0.60
PHA-1	Unadjusted	0.12	0.44	0.24	0.13
	Adjusted^a^	0.25	0.17	0.32	0.076
PHA-2	Unadjusted	-0.12	0.45	0.00	0.99
	Adjusted^a^	-0.05	0.79	0.01	0.94
Media-1	Unadjusted	-0.24	0.13	0.00	0.99
	Adjusted^a^	-0.37^b^	0.040^b^	-0.02	0.93
Media-2	Unadjusted	-0.25	0.10	-0.10	0.54
	Adjusted^a^	-0.38^b^	0.038^b^	-0.10	0.58

Shown are the Spearman correlation coefficients and associated *P*-values for the PCA stimulus-cytokine scores for the first and second principal components and both the change in the DAS28 and the change in HAQ from baseline to 21 to 24 weeks among 43 patients with early RA. ^a^Adjusted for age, sex, body mass index, smoking status, rheumatoid factor status, anti-citrullinated protein antibodies status, CMV immunoglobulin G status, methotrexate use, and prednisone use; ^b^values are statistically significant. ACR, American College of Rheumatology; DAS28, Disease Activity Score in 28 joints; CMV/EBV, cytomegalovirus/Epstein-Barr virus; PHA, phytohemagglutinin; HAQ-DI, Health Assessment Questionnaire disability index.

In contrast, the changes in both the media alone PCA-1 and PCA-2 scores were shown to correlate with the ΔDAS28 only after adjustment for the aforementioned covariates. Comparison of the media-alone PCA scores revealed no significant differences between responders and non-responders to DMARD treatment (Figure 2B). Pertinently, evidence of correlation with the ΔDAS28 was neither observed for the immune response profiles of PHA or CPG (Table 5), nor any of the other stimuli tested (data not shown).

### Correlative analysis of the CMV/EBV immune response with the change in clinical disease activity according to DMARD treatment

**Figure 3:**
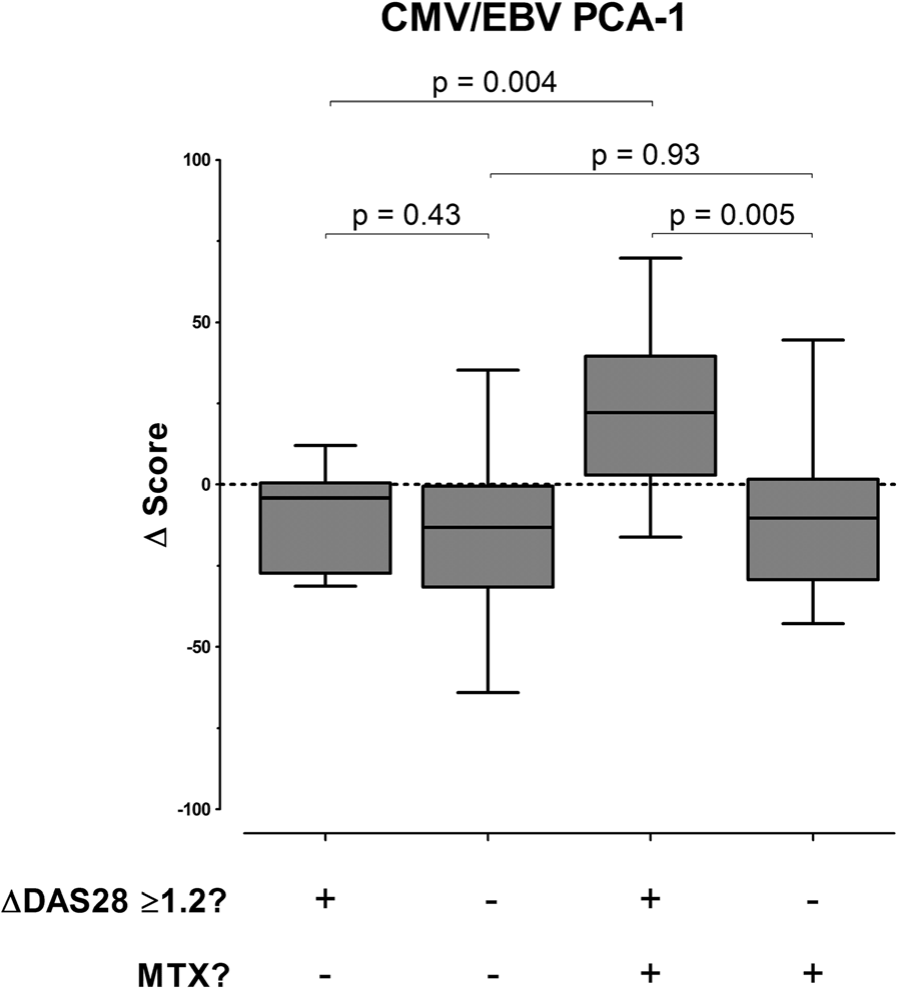
**Clinical improvement in the DAS28 related to methotrexate treatment compared to other disease-modifying antirheumatic drugs is uniquely associated with up-regulation of the cytomegalovirus/Epstein-Barr virus (CMV/EBV) immune-response signature.** Shown are box plots comparing the changes in the CMV/EBV PCA-1 score from baseline to 21 to 24 weeks of follow up among 43 patients with early rheumatoid arthritis among no MTX/non-responders (n = 13), no MTX/responders (n = 10), MTX/non-responders (n = 7), and MTX/responders (n = 13). The horizontal center lines indicate the medians, the boxes represent the interquartile ranges, and the whiskers depict the 2.5 and 97.5 percentiles. DAS28, disease activity score in 28 joints; PCA-1 score, principal components analysis-1 score; MTX, methotrexate.

Among the treatment non-responders, there was no evidence that DMARD therapy had any impact on the CMV/EBV PCA-1 immune response signature (Figure 3). However, among the patients who attained clinically meaningful DAS28 improvement, methotrexate use during the study was associated with a statistically significant increase in the CMV/EBV PCA-1 score from baseline to follow up (*P* = 0.004) (Figure 3). No significant associations between the change in CMV/EBV PCA-1 score and the use of methotrexate or corticosteroids were observed (data not shown). Although the baseline CMV/EBV PCA-1 score was correlated with the change in this score from baseline to follow up (*r* = -0.44), adjustment for the baseline CMV/EBV score did not change the relationship between methotrexate use and the changes in the CMV/EBV PCA-1 score.

### Exploration of the immune response profile for CMV/EBV

Having observed associations between the CMV/EBV immune-response profile and treatment response, we explored the relationships of this profile to clinical characteristics. The baseline CMV/EBV PCA-1 score did not correlate significantly with age, sex, disease duration, RF status, ACPA status, baseline DAS28, or baseline HAQ (data not shown). With regard to the potential for treatment effects on the CMV/EBV immune response, the mean CMV/EBV PCA-1 score at baseline was similar between prednisone users and non-users (61.3 versus 52.4, respectively; *P* = 0.2) and between patients who had and those who had not started taking DMARDs prior to baseline (58.7 versus 59.6, respectively; *P* = 0.78). Interestingly, the change in the CMV/EBV PCA-1 score from baseline to follow up was associated with RF positivity (median difference -16.1 in RF-negative versus -0.33 in RF-positive, *P* = 0.03). In contrast, the CMV/EBV PCA-2 score, which was not associated with treatment outcome, was associated with baseline DAS28 (*r* = 0.34; *P* = 0.004), baseline HAQ (*r* = 0.25; *P* = 0.04), and prior prednisone use (median 62.7 for no prior prednisone use versus 85.9 for prior prednisone use; *P* = 0.03).

**Figure 4:**
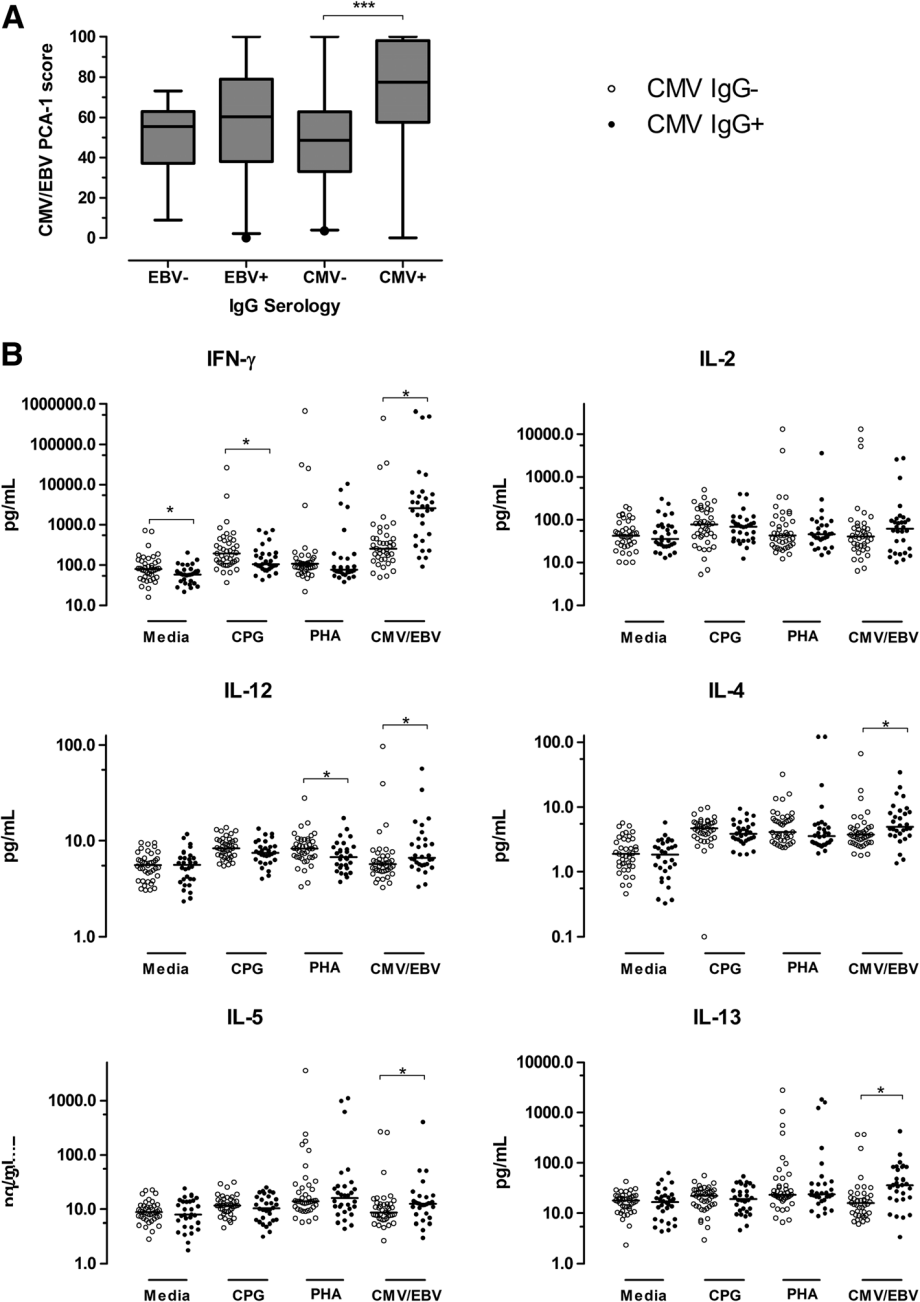
**Exposure to cytomegalovirus (CMV) is associated with the CMV/Epstein-Barr virus (EBV) immune-response signature as well as generally altered T-cell immunity. (A)** Box plots illustrate the distributions of the CMV/EBV PCA-1 score at baseline in 71 patients with RA, according to CMV and EBV serological status. The horizontal lines represent medians, the boxes represent the 25 to 75 percentiles, and the whiskers represent the 2.5 to 97.5 percentiles, and dots represent outliers. **(B)** Scatterplots depict the concentrations of selected cytokines (based on PCA weightings >0.5) for the immune-response profiles, according to CMV serological status and stimulus (CMV/EBV, PHA, CPG, and media alone) at baseline in 71 patients with RA. The horizontal bold lines represent the medians; ^*^P <0.05, ^***^P <0.001. RA, rheumatoid arthritis; DAS28, Disease Activity Score in 28 joints; PCA, principal components analysis; PHA, phytohemagglutinin; CPG, CpG oligonucleotides; IgG, immunoglobulin-G.

The CMV/EBV PCA-1 score, which represented the immune response to a mixture of viral lysates, was associated with CMV exposure (median 48.6 among CMV-IgG negative versus 76.8 among CMV-IgG positive; *P* <0.001; Figure 4A). In contrast, there was no association of this immune response with EBV IgG (median 55.4 for negative versus 60.4 for positive; *P* = 0.32) (Figure 4A). Of relevance was the lack of association between CMV-IgG status and the ΔDAS28 (mean difference -1.2 versus -1.5, *P* = 0.44).

Higher production of IFN-γ, IL-4, IL-5, IL-12, and IL-13 from PBMC in response to stimulation with CMV/EBV lysates was observed among CMV-IgG-positive patients as compared to CMV-IgG-negative patients (Figure 4B). In contrast, lower basal and CPG-induced production of IFN-γ as well as lower PHA-induced production of IL-12 was evident among CMV-IgG-positive patients as compared to CMV-IgG-negative patients. Otherwise, no other significant differences in the production of individual cytokines were observed for PHA or CPG (Figure 4B), or any of the other stimuli (data not shown).

## Discussion

We report the discovery of a T-cell immune-response signature associated with CMV immunity that is predictive of inadequate response to initial DMARD therapy among patients with early RA. Specifically, higher production of both type 1 (for example, IFN-γ) and type 2 (for example, IL-4) T-cell cytokines by PBMC, in response to stimulation with combined CMV/EBV lysate *ex vivo*, is predictive of inadequate DAS28 response to initial DMARD therapy over 21 to 24 weeks. Because the CMV/EBV immune-response signature was found to correlate to CMV IgG serology, the observed association with treatment response is likely mediated by CMV rather than EBV immunity. Further, we show that DMARD-induced amelioration of clinical disease activity correlates with augmentation of the CMV-related immune-response signature.

With further development, this signature could potentially be useful as a predictive biomarker for individualizing the management of early RA. Because the patients with high baseline CMV responsiveness experienced inferior outcomes of initial DMARD therapy, future studies should investigate whether a more aggressive treatment strategy in these patients could lead to more favorable outcomes. For example, the hypothesis could be tested that patients with the high CMV-specific T-cell immune-response signature would have better clinical response to therapy combining methotrexate and a TNF antagonist. The significance of this scenario is highlighted by the knowledge that 50 to 70% of patients fail to respond to initial methotrexate monotherapy [2,24], yet currently there are no biomarkers or prediction models that reliably and accurately identify these individuals [24-27]. The advent of new techniques for individualizing initial therapy could improve outcomes for patients with RA, by inducing clinical remission earlier and/or by sparing patients trials of costly and risky medications to which they are pre-destined to respond unfavorably [28,29].

The reported immune-response signature clearly requires further refinement and validation. First, we must verify that CMV is the target of the immune response in our signature that is predictive of treatment outcome, by evaluating profiles of cytokine response to CMV and EBV separately. Removing EBV from the CMV immune response assay could conceivably reduce both technical and biological variability in the response profile, resulting in a more robust and informative assay. Second, we must determine if this signature is associated with the clinical response to specific DMARDs or whether it is predictive of response to disease-modifying therapy in general. In this regard, a limitation of this study is the heterogeneity among patients in the selection of DMARD therapy. Therefore, it will be of interest to determine if the CMV-related immune-response signature is predictive of successful response to treatment with a TNF antagonist or other biologic agent using a defined protocol. Third, we recognize that the CMV-related immune-response signature as undertaken in this study would be challenging to translate to clinical settings, so further work is necessary to develop a practical, reliable, and scalable assay based on our approach. These crucial studies are currently underway at our institution.

The findings of this study imply that CMV-related T-cell immunity may interact with the pathophysiology of RA. A potential unifying hypothesis for our findings must take into account not only that higher baseline CMV immune responsiveness is predictive of inadequate DMARD treatment response, but also that increasing CMV immune responsiveness from baseline to follow up is associated with good clinical response to DMARD therapy. Previous studies have demonstrated that patients with RA often have impairments of systemic T-cell function. For example, production of IFN-γ or IL-2 in response to mitogens has generally been found to be significantly lower in patients with active RA compared to inactive RA or healthy control subjects [30-32]. In contrast, Pierer *et al*. have reported a significantly higher magnitude of CD4+ IFN-γ-secreting T cells in response to CMV pp65 or CMV lysate in patients with RA compared to controls [33]. Effective therapy for RA has been found to ameliorate the impaired T-cell responsiveness of IFN-γ production seen in RA patients [30,31,34]. However, our data suggest that the association between T-cell responsiveness and treatment outcome was specific for the CMV stimulus and not to other T-cell stimuli, including CD3/CD28, PHA, CPG, and PMA/ionomycin. Previous studies have observed the general phenomenon of RA T-cell hypo-responsiveness using these non-specific stimuli [30,35,36].

Rather, the findings of this study point to an interaction of rheumatoid disease specifically with CMV immunity. The significant correlation with CMV IgG suggests that the CMV-induced T-cell immune response signature is mediated by memory T cells. The nature of the CMV stimulus and pattern of T-cell cytokines suggest that this signature is mediated by CD4+ cells. Previous studies have shown that a subset of patients with RA has expanded pools of CMV-specific CD4+ IFN-γ-producing T cells in the peripheral blood [33,37,38]. The distribution of CD4+ memory T cells against CMV is skewed to higher frequencies of cells in the peripheral blood than synovial fluid [39]. These cells generally are thought to have a highly differentiated phenotype, lacking expression of the co-stimulatory molecule CD28, which has been associated with RA extra-articular manifestations and cardiovascular disease [40,41]. Positive CMV IgG and increased CMV-specific CD4+ CD28^null^ T cells have been reported to correlate with structural joint damage [33]. With respect to the relationship between improvement in clinical disease activity and the augmentation of the CMV response, our data suggest that attenuation of inflammation affects the systemic CMV-specific memory T-cell pool. Speculatively, this could arise due to changes in the number, function, or phenotype of CMV-specific memory T cells circulating in the blood [42,43]. Due to the limitations of this discovery-oriented study, future research is needed to clarify the underlying immune mechanisms and implications of our findings.

We report that among patients who were treated with methotrexate during follow up and who attained clinically meaningful DAS28 improvement, the CMV-related T-cell immune response signature was observed to increase significantly from baseline to follow up. In contrast, patients who were treated with non-methotrexate DMARDs or who did not achieve a meaningful DAS28 response generally had a decline in their CMV-associated T-cell response. The mechanism of this finding is unclear, and the observational design of this study precludes causal assessment of treatment effects. However, a possible implication is that various DMARDs may have different effects on CMV T-cell responses. Such heterogeneity of treatment effects on CMV immunity is potentially significant in view of the aforementioned association between CMV IgG and disease progression, suggesting some DMARDs may not sufficiently antagonize the contribution of CMV immunity to disease, leading to inadequate treatment response and disease progression.

## Conclusion

We have reported a novel interaction between the clinical response to initial DMARD therapy and an *ex vivo* immune response to CMV in patients with early RA. Together with our published data and findings from recent literature, the results of this study contribute new insights into the role of a T-cell response associated with CMV exposure in modulating the outcomes of DMARD treatment in early RA. The roles of subclinical CMV persistence and CMV-associated T-cell immunity in modulating the outcomes of RA deserve further investigation.

## Abbreviations

ACPA: Anti-citrullinated protein antibodies; ACR: American College of Rheumatology; CMV: Cytomegalovirus; CRP: C-reactive protein; DAS28: Disease Activity Score using 28 joints; DMARD: Disease-modifying antirheumatic drug; EBV: Epstein-Barr virus; G-CSF: Granulocyte colony stimulating factor; GM-CSF: Granulocyte monocyte colony stimulating factor; HAQ: Health assessment questionnaire; IFN: Interferon; Ig: Immunoglobulin; IL: Interleukin; MCP: Monocyte chemoattractant protein; MIP: Monocyte inflammatory protein; PBMC: Peripheral blood mononuclear cells; PCA: Principal components analysis; PHA: Phytohemagglutinin; PMA: Phorbol myristate acetate; RA: Rheumatoid arthritis; RF: Rheumatoid factor; SEA: Staphylococcal enterotoxin A; SEB: Staphylococcal enterotoxin B; TNF: Tumor necrosis factor.

## Competing interests

Mayo Foundation has submitted a patent application for the technique of multi-cytokine response profiling described in this article for assessing RA outcomes. JMD, KLK, and SEG are listed as inventors of technology in that patent application.

## Authors’ contributions

JMD obtained research funding, designed the study and analysis plan, recruited all patients, directed data interpretation, and primarily drafted and revised the manuscript. KLK conceived of the immune response profiling methodology, contributed to study design, and participated in data interpretation and manuscript preparation. MAS performed all immune-response profiling assays. ABG, CSC, and TMT performed the statistical analysis. ELM and SEG contributed to the study design, data analysis and interpretation, and manuscript revision. All authors have critically analyzed and approved the final submitted version of the manuscript.
